# A Robust Adaptive Filter for a Complex Hammerstein System

**DOI:** 10.3390/e21020162

**Published:** 2019-02-09

**Authors:** Guobing Qian, Dan Luo, Shiyuan Wang

**Affiliations:** 1 College of Electronic and Information Engineering, Chongqing Key Laboratory of Nonlinear Circuits and Intelligent Information Processing, Southwest University, Chongqing 400715, China; 2 School of Mathematics and Statistics, Southwest University, Chongqing 400715, China

**Keywords:** complex, Hammerstein, adaptive filters, impulsive noise, stability

## Abstract

The Hammerstein adaptive filter using maximum correntropy criterion (MCC) has been shown to be more robust to outliers than the ones using the traditional mean square error (MSE) criterion. As there is no report on the robust Hammerstein adaptive filters in the complex domain, in this paper, we develop the robust Hammerstein adaptive filter under MCC to the complex domain, and propose the Hammerstein maximum complex correntropy criterion (HMCCC) algorithm. Thus, the new Hammerstein adaptive filter can be used to directly handle the complex-valued data. Additionally, we analyze the stability and steady-state mean square performance of HMCCC. Simulations illustrate that the proposed HMCCC algorithm is convergent in the impulsive noise environment, and achieves a higher accuracy and faster convergence speed than the Hammerstein complex least mean square (HCLMS) algorithm.

## 1. Introduction

Since traditional mean square error (MSE) criterion derived algorithms are sensitive to outliers, they cannot be used to deal with impulsive noise effectively [1,2]. However, impulsive noise commonly exists in practice. To solve this problem, a higher-order statistic, named correntropy, was proposed [3,4]. It has been proven that the maximum correntropy criterion (MCC) algorithm is robust to impulsive noises, and outperforms the traditional MSE algorithms obviously when the noises are non-Gaussian distributed. Thus, the MCC algorithm [5,6] and its variants [7,8,9,10], such as generalized maximum correntropy criterion (GMCC) [7], are widely used in practice.

Different from the well-known Wiener adaptive filter [11], the Hammerstein adaptive filter consists of two parts, namely: a nonlinear memoryless polynomial function and a linear finite impulse response (FIR) filter [12,13,14]. The Hammerstein system has been widely applied to signal processing [15,16,17,18] as well as other applications [19,20]. Considering that the performance of the Hammerstein adaptive filter using MSE criterion decreases dramatically when an impulsive noise exists, Wu et al. applied the MCC criterion to the Hammerstein adaptive filter, and developed a robust Hammerstein adaptive filtering algorithm [21]. This novel adaptive filter is insensitive to outliers and behaves better than the traditional Hammerstein adaptive filters, especially in the case of the impulsive noise.

However, the Hammerstein adaptive filters under the traditional MSE criterion and MCC criterion are defined in the field of real numbers. They cannot be directly employed to handle the complex-valued data. In fact, many signals are defined in the complex domain in practical applications [22,23,24,25]. Thus, in this work, we put forward a Hammerstein maximum complex correntropy criterion (HMCCC) algorithm, which extends the Hammerstein adaptive filter, using MCC criterion, to the complex domain. HMCCC can be used to handle complex-valued data directly, while being robust to impulsive noise. We analyzed the stability and provided the steady-state mean square performance of the HMCCC algorithm. Simulations show that the HMCCC is robust to outliers, and achieves a higher accuracy and faster convergence speed than the Hammerstein complex least mean square (HCLMS) algorithm. 

The rest of the paper is organized as follows. A complex Hammerstein adaptive filter under MCCC is developed in Section 2. In Section 3, we analyze the stability and provide the steady-state mean square performance of the HMCCC algorithm. In Section 4, several simulations are presented so as to verify the superior performance of the HMCCC algorithm. Finally, a conclusion is drawn in Section 5.

## 2. Hammerstein Adaptive Filter under the Maximum Complex Correntropy Criterion

### 2.1. Complex Correntropy

Considering two complex variables that are $C_{1}=X_{1}+jY_{1}$ and $C_{2}=X_{2}+jY_{2}$, respectively, the complex correntropy is defined by the following [22]:

(1) $$V_{}^{c}\left(C_{1},C_{2}\right)=E\left[\kappa_{\sigma }^{c}\left(C_{1}-C_{2}\right)\right]$$

where $\kappa_{\sigma }^{c}\left(C_{1}-C_{2}\right)$ represents the kernel function, and $X_{1}$, $Y_{1}$, $X_{2}$, and $Y_{2}$ denote the real variables.

A Gaussian kernel is adopted in this paper, which is expressed as follows:

(2) $$\kappa_{\sigma }^{c}\left(C_{1}-C_{2}\right)=\frac{1}{2\pi \sigma^{2}}\exp\left(-\frac{\left(C_{1}-C_{2}\right){\left(C_{1}-C_{2}\right)}^{*}}{2\sigma^{2}}\right)$$

with σ being the kernel width.

### 2.2. Adaptive Filter for Complex Hammerstein System

#### 2.2.1. Cost Function 

Consider a complex Hammerstein system, the output of polynomial nonlinear part is as follows:

(3) $$s\left(k\right)=p^{H}x_{p}\left(k\right)$$

where $p={\left[\begin{array}{cccc} p_{1} & p_{2} & \cdots & p_{M} \end{array}\right]}^{T}$ denotes the vector of the complex polynomial coefficient, M is the polynomial order, and $x_{p}\left(k\right)={\left[\begin{array}{cccc} x\left(k\right) & x^{2}\left(k\right) & \cdots & x^{M}\left(k\right) \end{array}\right]}^{T}$ is the complex polynomial regressor vector, ${\left(\cdot \right)}^{T}$ and ${\left(\cdot \right)}^{H}$ denote the transpose and conjugate transpose, respectively.

The cost function of the complex Hammerstein filtering algorithm under MCCC is as follows:

(4) $$J_{HMCCC}^{}=E\left[\kappa_{\sigma }^{c}\left(e\left(k\right)\right)\right]$$

where $e\left(k\right)=d\left(k\right)-w^{H}s\left(k\right)$ is the error at the k-th iteration, $w={\left[\begin{array}{cccc} w_{1} & w_{2} & \cdots & w_{N} \end{array}\right]}^{T}$ denotes the estimated weight vector, $s\left(k\right)=[s\left(k\right){\begin{array}{ccc} s\left(k-1\right) & \cdots & s\left(k-N+1\right) \end{array}]}^{T}$ is the input vector of the complex finite impulse response (FIR) filter, $d\left(k\right)=w_{0}^{H}s_{0}\left(k\right)+v\left(k\right)$ is the desired signal at the k-th iteration, $s_{0}\left(k\right)=[s_{0}\left(k\right){\begin{array}{ccc} s_{0}\left(k-1\right) & \cdots & s_{0}\left(k-N+1\right) \end{array}]}^{T}$, $s_{0}\left(k\right)=p_{0}^{H}x_{p}\left(k\right)$, N denotes the length of the linear FIR filter, $w_{0}$ and $p_{0}$ are the unknown system parameters to be estimated, which are the optimum solutions for w and p, and v(k) is the observation noise. 

#### 2.2.2. Adaptive Algorithm

Based on the Wirtinger Calculus [26,27,28], we derive the stochastic gradient of $J_{HMCCC}^{}$ with respect to $p^{*}$ as follows:

(5) $$\frac{\partial J_{HMCCC}^{}}{\partial p^{*}}=\frac{1}{2\pi \sigma^{2}}\exp\left[-\frac{{\left|e\left(k\right)\right|}^{2}}{2\sigma^{2}}\right]\frac{1}{2\sigma^{2}}e^{*}\left(k\right)X\left(k\right)w^{*}\left(k\right)$$

and with respect to $w^{*}$ as follows:

(6) $$\frac{\partial J_{HMCCC}^{}}{\partial w^{*}}=\frac{1}{2\pi \sigma^{2}}\exp\left[-\frac{{\left|e\left(k\right)\right|}^{2}}{2\sigma^{2}}\right]\frac{1}{2\sigma^{2}}e^{*}\left(k\right)X^{T}\left(k\right)p^{*}\left(k\right)$$

where $X\left(k\right)=\left[\begin{array}{cccc} x_{p}\left(k\right) & x_{p}\left(k-1\right) & \cdots & x_{p}\left(k-N+1\right) \end{array}\right]$.

Then, the updates for p and w are as follows:

(7) $$p\left(k+1\right)=p\left(k\right)+\eta_{p}f\left(e\left(k\right)\right)X\left(k\right)w^{*}\left(k\right)$$

(8) $$w\left(k+1\right)=w\left(k\right)+\eta_{w}f\left(e\left(k\right)\right)X^{T}\left(k\right)p^{*}\left(k\right)$$

where $f\left(e\left(k\right)\right)=\exp\left[-\frac{{\left|e\left(k\right)\right|}^{2}}{2\sigma^{2}}\right]e^{*}\left(k\right)$ and the constant $\frac{1}{2\pi \sigma^{2}}$ is merged into the step-size parameters $\eta_{p}$ and $\eta_{w}$.

Finally, Table 1 summarizes the HMCCC algorithm. 

## 3. Convergence Analysis

To begin the derivation of the convergence analysis, some widely used assumptions are adopted, as follows:

(A1) v(k) is independently identically distributed (iid), zero-mean, circular, and independent of $e_{p}\left(k\right)$, $e_{w}\left(k\right)$ and x(k);
(A2) Both ${\left\|X^{T}\left(k\right)p^{*}\left(k\right)\right\|}^{2}$ and ${\left\|X\left(k\right)w^{*}\left(k\right)\right\|}^{2}$ are uncorrelated with ${\left|f\left(e\left(k\right)\right)\right|}^{2}$ when k→∞.

**Remark** **1:** *(1) A1 is reasonable in a practical case and is widely used in the theoretical analysis of an adaptive filter [21,29,30];*

*(2) When*k→∞*,*$p\left(k\right)\to p_{0}\left(k\right)$*and*$w\left(k\right)\to w_{0}\left(k\right)$*. Additionally,*X(k)*is independent of*e(k)*, based on A1. Thus, both*${\left\|X^{T}\left(k\right)p^{*}\left(k\right)\right\|}^{2}$*and*${\left\|X\left(k\right)w^{*}\left(k\right)\right\|}^{2}$*are uncorrelated with*${\left|f\left(e\left(k\right)\right)\right|}^{2}$. *Thus, A2 is also a reasonable assumption.*

### 3.1. Stability Analysis

As ${\left|e\left(k\right)\right|}^{2}$ is a real-valued function for ${\left[\begin{array}{cc} p^{T}\left(k\right) & w^{T}\left(k\right) \end{array}\right]}^{T}$, the following expression can be derived by taking the Taylor series expansion of ${\left|e\left(k+1\right)\right|}^{2}$ at ${\left[\begin{array}{cc} p^{T}\left(k\right) & w^{T}\left(k\right) \end{array}\right]}^{T}$,

(9) |e(k+1)|2=|e(k)|2+2Re{∂|e(k)|2∂p∗(k)|w(k)=cons⋅(Δp(k))∗}+2Re{∂|e(k)|2∂w∗(k)|p(k)=cons⋅(Δw(k))∗}+h.o.t.

where

(10) $$\frac{\partial {\left|e\left(k\right)\right|}^{2}}{\partial p^{*}\left(k\right)}=-X\left(k\right)w^{*}\left(k\right)e^{*}\left(k\right)$$

(11) $$\frac{\partial {\left|e\left(k\right)\right|}^{2}}{\partial w^{*}\left(k\right)}=-X^{T}\left(k\right)p^{*}\left(k\right)e^{*}\left(k\right)$$

(12) $$\Delta p\left(k\right)=\eta_{p}f\left(e\left(k\right)\right)X\left(k\right)w^{*}\left(k\right)$$

(13) $$\Delta w\left(k\right)=\eta_{w}f\left(e\left(k\right)\right)X^{T}\left(k\right)p^{*}\left(k\right)$$

and h.o.t represents the terms of higher order infinitesimal. 

Then,

(14) $$\begin{array}{cc} E\left\{{\left|e\left(k+1\right)\right|}^{2}\right\} & =E\left\{\left[1-2\eta_{p}\exp\left[-\frac{{\left|e\left(k\right)\right|}^{2}}{2\sigma^{2}}\right]{\left\|X\left(k\right)w^{*}\left(k\right)\right\|}^{2}-2\eta_{w}\exp\left[-\frac{{\left|e\left(k\right)\right|}^{2}}{2\sigma^{2}}\right]{\left\|X^{T}\left(k\right)p^{*}\left(k\right)\right\|}^{2}\right]{\left|e\left(k\right)\right|}^{2}\right\}\\ & \approx E\left\{\left[1-2\eta_{p}\exp\left[-\frac{{\left|e\left(k\right)\right|}^{2}}{2\sigma^{2}}\right]{\left\|X\left(k\right)w^{*}\left(k\right)\right\|}^{2}-2\eta_{w}\exp\left[-\frac{{\left|e\left(k\right)\right|}^{2}}{2\sigma^{2}}\right]{\left\|X^{T}\left(k\right)p^{*}\left(k\right)\right\|}^{2}\right]\right\}E\left\{{\left|e\left(k\right)\right|}^{2}\right\} \end{array}$$

Thus, the sequence |e(k)| will decrease in the mean sense if

(15) $$E\left\{\left|1-2\eta_{p}\exp\left[-\frac{{\left|e\left(k\right)\right|}^{2}}{2\sigma^{2}}\right]{\left\|X\left(k\right)w^{*}\left(k\right)\right\|}^{2}-2\eta_{w}\exp\left[-\frac{{\left|e\left(k\right)\right|}^{2}}{2\sigma^{2}}\right]{\left\|X^{T}\left(k\right)p^{*}\left(k\right)\right\|}^{2}\right|\right\}\leq 1$$

that is,

(16) $$0\leq E\left\{\left|\eta_{p}{\left\|X\left(k\right)w^{*}\left(k\right)\right\|}^{2}+\eta_{w}{\left\|X^{T}\left(k\right)p^{*}\left(k\right)\right\|}^{2}\right|\right\}\leq \frac{1}{E\left\{\exp\left[-\frac{{\left|e\left(k\right)\right|}^{2}}{2\sigma^{2}}\right]\right\}}$$

Considering the fact that $\exp\left[-\frac{{\left|e\left(k\right)\right|}^{2}}{2\sigma^{2}}\right]\leq 1$, we can obtain that the sequence |e(k)| will decrease in the mean sense, if

(17) $$0\leq E\left[\left|\eta_{p}{\left\|X\left(k\right)w^{*}\left(k\right)\right\|}^{2}+\eta_{w}{\left\|X^{T}\left(k\right)p^{*}\left(k\right)\right\|}^{2}\right|\right]\leq 1$$

In this case, the HMCCC algorithm will converge in the mean sense.

### 3.2. Steady Excess Mean Square Error

We define $H_{p}=\underset{k\to \infty }{\lim}E\left[{\left|e_{p}\left(k\right)\right|}^{2}\right]$ as the steady excess mean square error (EMSE) for the nonlinear part, $H_{w}=\underset{k\to \infty }{\lim}E\left[{\left|e_{w}\left(k\right)\right|}^{2}\right]$ as the steady EMSE for the linear filter, and $H_{pw}=\underset{k\to \infty }{\lim}E\left[{\left|e_{pw}\left(k\right)\right|}^{2}\right]$ as the steady EMSE for the whole Hammerstein system, where $e_{p}\left(k\right)=w_{0}^{H}X^{T}\left(k\right)p_{0}^{*}-w_{0}^{H}\left(k\right)X^{T}\left(k\right)p_{}^{*}\left(k\right)$, $e_{w}\left(k\right)=w_{0}^{H}X^{T}\left(k\right)p_{0}^{*}\left(k\right)-w_{}^{H}\left(k\right)X^{T}\left(k\right)p_{0}^{*}\left(k\right)$, $e_{pw}\left(k\right)=w_{0}^{H}X^{T}\left(k\right)p_{0}^{*}-w_{}^{H}\left(k\right)X^{T}\left(k\right)p_{}^{*}\left(k\right)$. 

When the algorithm reaches the steady, the error for the whole Hammerstein system can be approximately divided into two parts, as follows:

(18) $$\begin{array}{ll} e_{pw}\left(k\right) & =w_{0}^{H}X^{T}\left(k\right)p_{0}^{*}-w_{}^{H}\left(k\right)X^{T}\left(k\right)p_{}^{*}\left(k\right)\\ & =w_{0}^{H}X^{T}\left(k\right)p_{0}^{*}-w_{0}^{H}X^{T}\left(k\right)p_{}^{*}\left(k\right)+w_{0}^{H}X^{T}\left(k\right)p_{}^{*}\left(k\right)-w_{}^{H}\left(k\right)X^{T}\left(k\right)p_{}^{*}\left(k\right)\\ & \approx w_{0}^{H}\left(k\right)X^{T}\left(k\right){\tilde{p}}_{}^{*}\left(k\right)+{\tilde{w}}_{}^{H}\left(k\right)X^{T}\left(k\right)p_{0}^{*}\left(k\right)\\ & =e_{p}\left(k\right)+e_{w}\left(k\right) \end{array}$$

When only the nonlinear part is taken into consideration, we have

(19) $$\begin{array}{ll} e_{p}\left(k\right) & =w_{0}^{H}X^{T}\left(k\right)p_{0}^{*}-w_{0}^{H}\left(k\right)X^{T}\left(k\right)p_{}^{*}\left(k\right)\\ & \approx w_{0}^{H}\left(k\right)X^{T}\left(k\right){\tilde{p}}_{}^{*}\left(k\right) \end{array}$$

and

(20) $$\tilde{p}\left(k+1\right)=\tilde{p}\left(k\right)-\eta_{p}f\left(e\left(k\right)\right)X\left(k\right)w_{0}^{*}\left(k\right)$$

Multiplying each side of Equation (20) by its conjugate transpose and taking the expectation, we obtain the following:

(21) E{‖p˜(k+1)‖2}=E{‖p˜(k)‖2}−2ηpE{Re[ep(k)f(e(k))]}+ηp2E{‖X(k)w0∗(k)‖2|f(e(k))|2}

Since $\underset{k\to \infty }{\lim}E\left\{{\left\|\tilde{p}\left(k+1\right)\right\|}^{2}\right\}=\underset{k\to \infty }{\lim}E\left\{{\left\|\tilde{p}\left(k\right)\right\|}^{2}\right\}$, we further obtain the following:

(22) 2limk→∞E{Re[ep(k)f(e(k))]}=limk→∞ηpE{‖X(k)w0∗(k)‖2|f(e(k))|2}

Based on the results of Equations (38) and (46) in the literature [23], we similarly obtain the following expressions by replacing α and λ with 1 and $1/2\sigma^{2}$, respectively, as follows:

(23) E{Re[ep(k)f(e(k))]}=HpE{exp[−|v(k)|2/2σ2][1−(|v(k)|2/2σ2)]}

(24) $$E\left\{{\left|f\left(e\left(k\right)\right)\right|}^{2}\right\}=E\left\{\exp\left[-{\left|v\left(k\right)\right|}^{2}/\sigma^{2}\right]{\left|v\left(k\right)\right|}^{2}\right\}+H_{p}\times R_{1}$$

where

(25) $$R_{1}=E\{\exp\left[-{\left|v\left(k\right)\right|}^{2}/\sigma^{2}\right]\left[{\left|v\left(k\right)\right|}^{4}/\sigma^{4}-3{\left|v\left(k\right)\right|}^{2}/\sigma^{2}-1\right]\}$$

Furthermore, based on the result of Equation (47) in the literature [23], we obtain the EMSE for the nonlinear part by replacing $Tr\left(R_{xx^{H}}\right)$ (i.e., $E\left\{{\left\|x\left(i\right)\right\|}^{2}\right\}$) with $E\left\{{\left\|X\left(k\right)w_{0}^{*}\right\|}^{2}\right\}$,

(26) $$H_{p}=\frac{\eta_{p}^{}E\left\{{\left\|X\left(k\right)w_{0}^{*}\right\|}^{2}\right\}E\left\{\exp\left[-{\left|v\left(k\right)\right|}^{2}/\sigma^{2}\right]{\left|v\left(k\right)\right|}^{2}\right\}}{2E\left\{\exp\left[-{\left|v\left(k\right)\right|}^{2}/2\sigma^{2}\right]\left[1-\left({\left|v\left(k\right)\right|}^{2}/2\sigma^{2}\right)\right]\right\}-\eta_{p}^{}E\left\{{\left\|X\left(k\right)w_{0}^{*}\right\|}^{2}\right\}R_{1}}$$

When only the FIR filter is taken into consideration, we similarly derive

(27) $$H_{w}=\frac{\eta_{w}^{}E\left\{{\left\|X^{T}\left(k\right)p_{0}^{*}\right\|}^{2}\right\}E\left\{\exp\left[-{\left|v\left(k\right)\right|}^{2}/\sigma^{2}\right]{\left|v\left(k\right)\right|}^{2}\right\}}{2E\left\{\exp\left[-{\left|v\left(k\right)\right|}^{2}/2\sigma^{2}\right]\left[1-\left({\left|v\left(k\right)\right|}^{2}/2\sigma^{2}\right)\right]\right\}-\eta_{w}^{}E\left\{{\left\|X^{T}\left(k\right)p_{0}^{*}\right\|}^{2}\right\}R_{1}}$$

Furthermore, when both the nonlinear part and FIR filter are considered, we have

(28) Hpw=limk→∞E[|epw(k)|2]≈limk→∞E[|ep(k)+ew(k)|2]=limk→∞E[|ep(k)|2]+limk→∞E[|ew(k)|2]+limk→∞E[2Re(ep∗(k)ew(k))]=Hp+Hw+Hcross

**Remark** **2:** Hcross=limk→∞E[2Re(ep∗(k)ew(k))] *is the cross EMSE of the Hammerstein system, and equals zero when both* $e_{p}\left(k\right)$ *and* $e_{w}\left(k\right)$ *are zero mean and independent.*

## 4. Simulation

In this part, we provide some simulations to illustrate the superior performance of HMCCC. We chose the weight vector as $w_{0}={\left[\begin{array}{cccccc} 1+0.6j & 0.6+j & 0.1+0.2j & 0.2+0.1j & 0.06+0.04j & 0.04+0.06j \end{array}\right]}^{T}$ and the complex polynomial coefficient vector as $p_{0}={\left[\begin{array}{cc} 1+0.6j & 0.6+j \end{array}\right]}^{T}$. An additive complex noise $v=v_{R}+jv_{I}$ was considered in the simulations, with $v_{R}$ and $v_{I}$ being the real and imaginary parts, respectively. We compared the performance of HMCCC with HCLMS (HCLMS is the extension of HLMS [17] to complex domain, which is summarized in the Appendix A), and chose the parameters of both algorithms by trial, in order to ensure a desirable solution. Simulation results were obtained by averaging 100 Monte Carlo trials. The input signal x(k) was generated by a first-order autoregressive process, as follows:

(29) $$x\left(k\right)=ax\left(k-1\right)+\sqrt{1-a^{2}}\xi \left(k\right)$$

where $x\left(k\right)=x_{R}\left(k\right)+jx_{I}\left(k\right)$, $x_{R}\left(k\right)$, and $x_{I}\left(k\right)$ are the real and imaginary parts of x(k), a=0.95, $\xi \left(k\right)=\xi_{R}\left(k\right)+j\xi_{I}\left(k\right)$, $\xi_{R}\left(k\right)$, and $\xi_{I}\left(k\right)$ are the real and imaginary parts of ξ(k), $\xi_{R}\left(k\right),\xi_{I}\left(k\right)∼N\left(0,1\right)$, and $N\left(\mu ,\sigma^{2}\right)$ denotes the Gaussian distribution with mean μ and variance $\sigma^{2}$, respectively.

**Figure 1:**
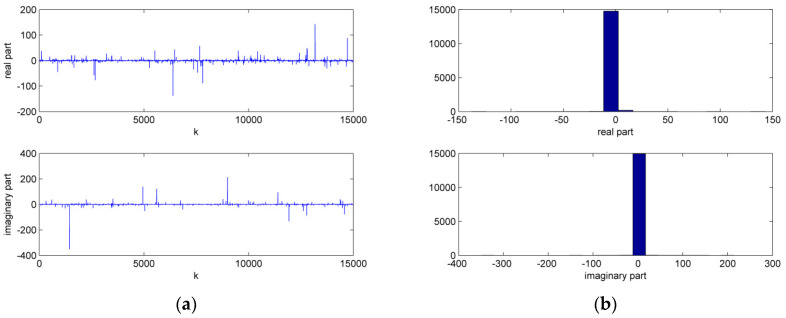
Time sequence and histogram for the complex alpha stable noise. (**a**) time sequence; (**b**) histogram.

**Figure 2:**
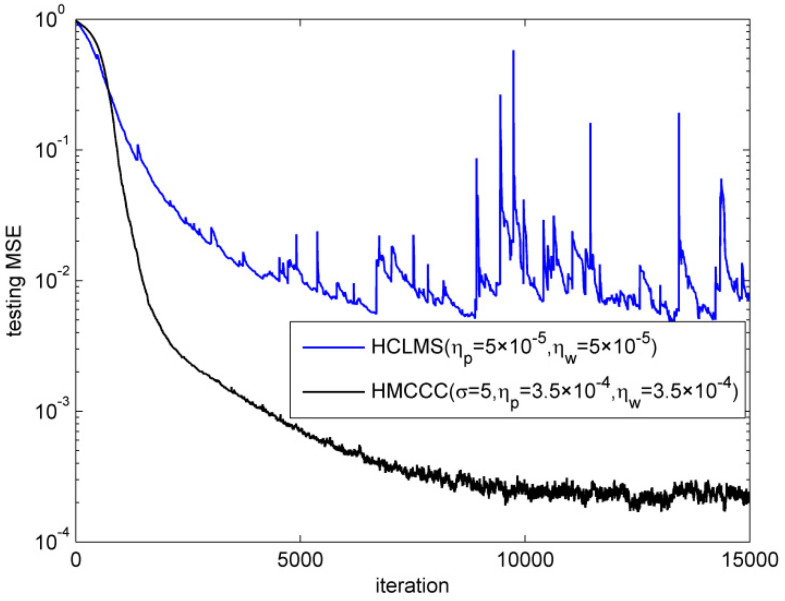
Learning curves of different algorithms. HCLMS—Hammerstein complex least mean square; HMCCC—Hammerstein maximum complex correntropy criterion.

First, the superiority of HMCCC was verified in the complex alpha stable noise environment. The noise parameters were $v_{R},v_{I}\in \sigma_{v}\cdot v_{alpha}\left(\alpha ,\beta ,\gamma ,\delta \right)$, where $\sigma_{v}^{2}=0.1$, α=1.2 is the characteristic factor, β=0 is the symmetry parameter, γ=0.6 is the dispersion parameter, and δ=0 is the location parameter, respectively. Figure 1 shows the time sequence and histogram for the real and imaginary parts of the complex alpha stable noise. It is noted that HCLMS may diverge in the complex alpha stable noise environment. Thus, we omitted the trials for HCLMS if ${\left\|w\right\|}_{2}\geq 100$. The simulation shows that HCLMS diverged twice in the 100 trials, while HMCCC did not diverge. The performances of the different algorithms in terms of the normalized testing mean square error (MSE) are shown in Figure 2, where the testing MSE was obtained from a test set of 100 samples, and the trials of the divergence were omitted for HCLMS. It is clear that compared with HCLMS, HMCCC has a better filtering performance in the presence of complex alpha stable noise.

**Figure 3:**
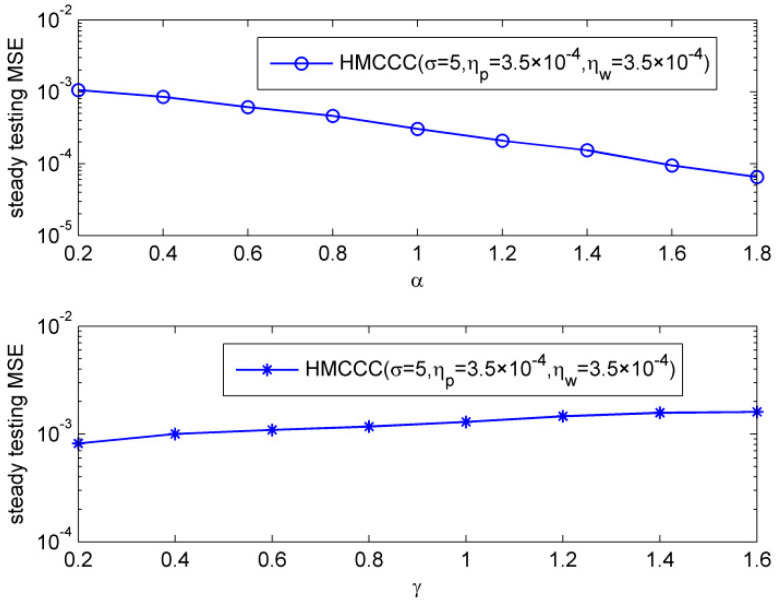
Steady testing mean square errors (MSEs) under different characteristic factors and dispersion parameters.

Then, we compared the steady testing MSE of HMCCC under different noise parameters. We ran 15,000 iterations to make sure the HMCCC reaches steady, and calculated the steady testing MSE with the average of next 1000 iterations. Figure 3 shows the steady normalized testing MSEs under different characteristic factors and dispersion parameters, respectively. It illustrates that HMCCC can perform well under different parameters of alpha stable noise.

**Figure 4:**
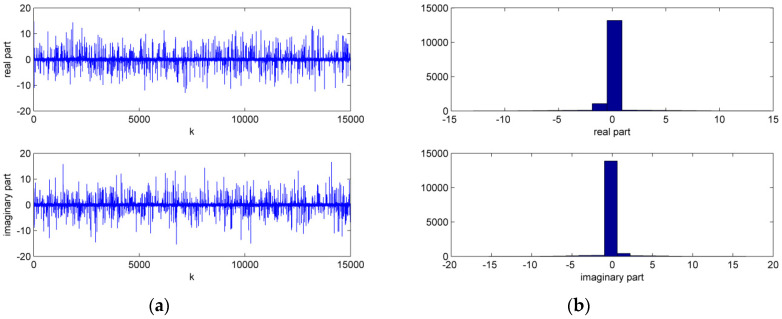
Time sequence and histogram for the contaminated Gaussian (CG) noise. (**a**) time sequence; (**b**) histogram.

**Figure 5:**
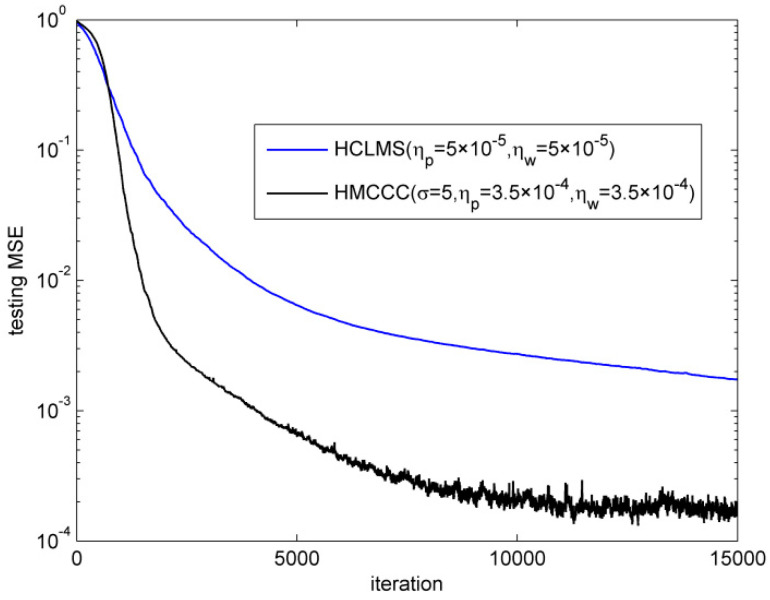
Learning curves of different algorithms.

Next, the superiority of HMCCC is verified in the contaminated Gaussian (CG) noise environment, where $v\left(k\right)=\left(1-c\left(k\right)\right)v_{1}\left(k\right)+c\left(k\right)v_{2}\left(k\right)$, $v_{1}\left(k\right)=v_{1R}\left(k\right)+jv_{1I}\left(k\right)$, $v_{2}\left(k\right)=v_{2R}\left(k\right)+$ $jv_{2I}\left(k\right)$, $v_{1R},v_{1I}\in N\left(0,0.1\right)$, and $v_{2R},v_{2I}\in N\left(0,20\right)$ represent outliers. Additionally,P(c(k)=1)=0.06, P(c(k)=0)=1−0.06. Figure 4 shows the time sequence and histogram for the real and imaginary parts of the CG noise. The performances of the different algorithms on the basis of normalized testing MSE are shown in Figure 5, where the testing MSE was also obtained from a test set of 100 samples. One can clearly see that, compared with HCLMS, HMCCC has a better filtering performance in the presence of CG noise. 

**Figure 6:**
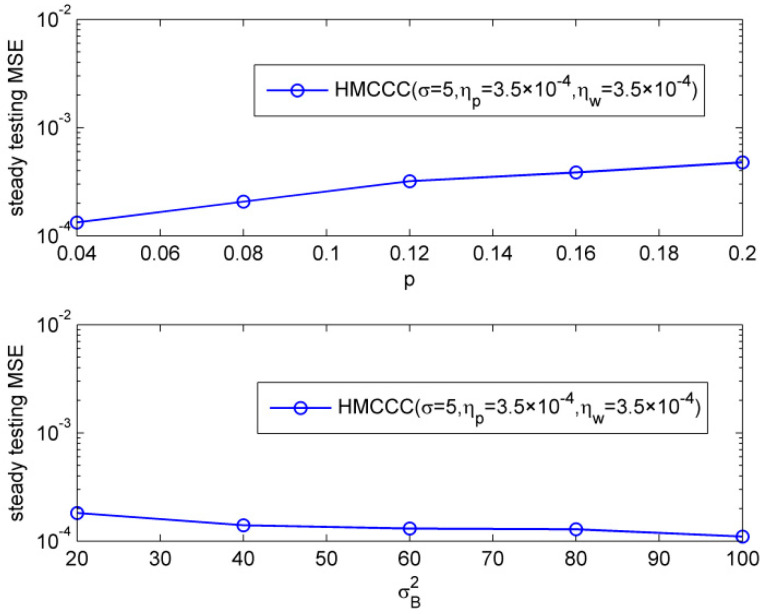
Influence of the probability and variance of outlier.

Furthermore, we tested the robustness of the HMCCC algorithm to the outlier. The CG noise was also used in this simulation, where $v_{1R},v_{1I}\in N\left(0,0.1\right)$, P(c(k)=1)=p, $v_{2R},v_{2I}\in N\left(0,\sigma_{B}^{2}\right)$, and P(c(k)=0)=1−p. Figure 6 depicts the steady normalized testing MSE of the HMCCC algorithm in the case of different probabilities of outlier, p ($\sigma_{B}^{2}=20$), and variances of outlier, $\sigma_{B}^{2}\left(p=0.06\right)$, where 15,000 iterations were run to make sure HMCCC reached steady, and the steady normalized testing MSEs were calculated with the average of the next 1000 iterations. One can observe that the proposed HMCCC algorithm is robust to the outlier, and behaves well under different p and $\sigma_{B}^{2}$. Moreover, HMCCC has a slightly smaller steady testing MSE with a bigger $\sigma_{B}^{2}$, which is a little surprising, but is consistent with Chen’s work [9]. This is due to the fact that the convergence rates are slightly different under different $\sigma_{B}^{2}$, even with the same learning rate. 

**Figure 7:**
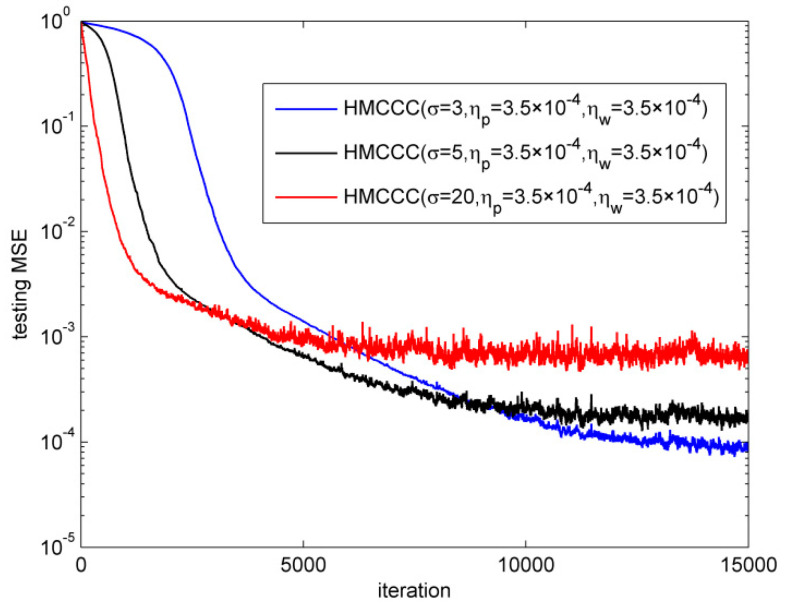
Influence of σ.

Afterward, we investigated the influences of the kernel width σ on the performance of HMCCC. The CG noise was also employed in this simulation, where $v_{1R},v_{1I}\in N\left(0,0.1\right)$, P(c(k)=1)=0.06, $v_{2R},v_{2I}\in N\left(0,20\right)$, and P(c(k)=0)=1−0.06. Figure 7 presents the normalized testing MSE of HMCCC in the case of three different kernel widths σ. It can be seen that kernel width σ has a vital role on the learning rate and steady value of HMCCC. With a small kernel width, HMCCC converges slowly, but achieves a small steady value. On the contrary, with a large kernel width, HMCCC converges quickly, but achieves a high steady value.

**Figure 8:**
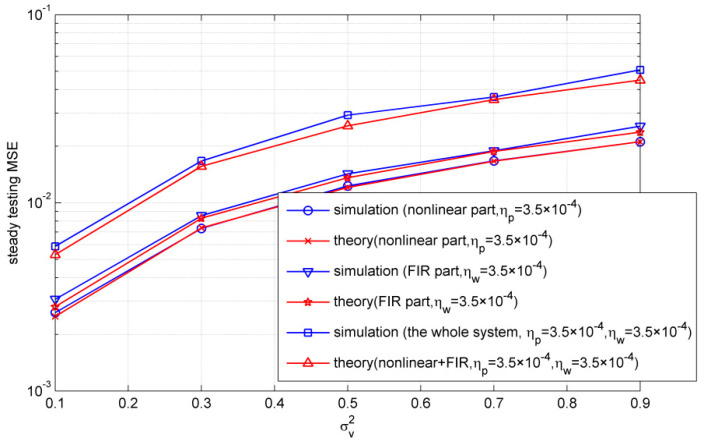
Steady-state testing MSEs with different noise variances (σ=5).

Finally, we compared the simulated steady testing MSEs with the theoretical ones. The Gaussian noise is used in this simulation, where $v\left(k\right)=v_{R}\left(k\right)+jv_{I}\left(k\right)$ and $v_{R},v_{I}\in N\left(0,\sigma_{v}^{2}\right)$. It is noted that the testing MSEs were not normalized in this simulation, and were obtained from a test set of 1000 samples. In addition, the theoretical values for the nonlinear part and FIR part were calculated by Equations (26) and (27), respectively. Figure 8 shows the simulated steady testing MSEs and the theoretical ones under different $\sigma_{v}^{2}$, where 40,000 iterations were run to make sure the algorithm reached steady, and the steady testing MSEs were calculated with the average of the next 1000 iterations. One can see that the simulated values almost matched with the theoretical ones for the nonlinear part and FIR part. Moreover, there is a little gap between the simulated whole system and the sum of theoretical nonlinear and FIR parts, which is the value of cross EMSE.

## 5. Conclusions

Since the Hammerstein adaptive filter can only be used to deal with real-valued data, in this paper, we extended the Hammerstein filter under maximum correntropy criterion (MCC) to the complex domain and developed a new algorithm, named the Hammerstein maximum complex correntropy criterion (HMCCC). Simultaneously, we analyzed the stability and derived some theoretical results for the HMCCC algorithm. The simulation illustrated that HMCCC is always convergent and performs better than the traditional Hammerstein complex LMS (HCLMS) algorithm in the presence of impulsive noises. Additionally, the kernel width has an important impact on the performance of HMCCC, and the novel algorithm behaves well with different probabilities and variances of outliers. 

## Figures and Tables

**Table 1:** The Hammerstein maximum complex correntropy criterion (HMCCC) algorithm.

**Input:** σ **,** $\eta_{p}$ **,** $\eta_{w}$ **,** M **,** N **,** d(k) **,** x(k)
1. Initializations: p(0), w(0).
2. While $\left\{\begin{array}{cc} d\left(k\right) & x\left(k\right) \end{array}\right\}$ available, do
3. $x_{p}\left(k\right)={\left[\begin{array}{cccc} x\left(k\right) & x^{2}\left(k\right) & \cdots & x^{M}\left(k\right) \end{array}\right]}^{T}$
4. $X\left(k\right)=\left[\begin{array}{cccc} x_{p}\left(k\right) & x_{p}\left(k-1\right) & \cdots & x_{p}\left(k-N+1\right) \end{array}\right]$
5. $s\left(k\right)=X^{T}\left(k\right)p^{*}\left(k\right)$
6. $e\left(k\right)=d\left(k\right)-w^{H}\left(k\right)s\left(k\right)$
7. $f\left(e\left(k\right)\right)=\exp\left[-\frac{{\left|e\left(k\right)\right|}^{2}}{2\sigma^{2}}\right]e^{*}\left(k\right)$
8. $p\left(k+1\right)=p\left(k\right)+\eta_{p}f\left(e\left(k\right)\right)X\left(k\right)w^{*}\left(k\right)$
9. $w\left(k+1\right)=w\left(k\right)+\eta_{w}f\left(e\left(k\right)\right)X^{T}\left(k\right)p^{*}\left(k\right)$
10. End while
11. ${\hat{w}}_{0}=w\left(k+1\right)$
Output: Estimated polynomial coefficient ${\hat{p}}_{0}$ and filter weight ${\hat{w}}_{0}$.

## Appendix A

The summary of the HCLMS algorithm:

**Input:** $\eta_{p}$ **,** $\eta_{w}$ **,** M **,** N **,** d(k) **,** x(k)
1. Initializations: p(0), w(0).
2. While $\left\{\begin{array}{cc} d\left(k\right) & x\left(k\right) \end{array}\right\}$ available, do
3. $x_{p}\left(k\right)={\left[\begin{array}{cccc} x\left(k\right) & x^{2}\left(k\right) & \cdots & x^{M}\left(k\right) \end{array}\right]}^{T}$
4. $X\left(k\right)=\left[\begin{array}{cccc} x_{p}\left(k\right) & x_{p}\left(k-1\right) & \cdots & x_{p}\left(k-N+1\right) \end{array}\right]$
5. $s\left(k\right)=X^{T}\left(k\right)p^{*}\left(k\right)$
6. $e\left(k\right)=d\left(k\right)-w^{H}\left(k\right)s\left(k\right)$
7. $p\left(k+1\right)=p\left(k\right)+\eta_{p}e^{*}\left(k\right)X\left(k\right)w^{*}\left(k\right)$
8. $w\left(k+1\right)=w\left(k\right)+\eta_{w}e^{*}\left(k\right)X^{T}\left(k\right)p^{*}\left(k\right)$
9 End while
10. ${\hat{w}}_{0}=w\left(k+1\right)$
Output: Estimated polynomial coefficient ${\hat{p}}_{0}$ and filter weight ${\hat{w}}_{0}$.
